# The Reliability and Validity of a New Laryngeal Palpation Tool for Static and Dynamic Examination

**DOI:** 10.3390/jcm14176309

**Published:** 2025-09-06

**Authors:** Isabelle Bargar, Melina Maria Ippers, Katrin Neumann, Philipp Mathmann, Ben Barsties v. Latoszek

**Affiliations:** 1 School of Health, Education and Social Sciences, SRH University of Applied Sciences Heidelberg, 40210 Düsseldorf, Germany; 2 Department of Phoniatrics and Pediatric Audiology, University Hospital Münster, 48149 Münster, Germany; 3 Department of Otorhinolaryngology, Head and Neck Surgery, Johannes Wesling Klinikum Minden, Ruhr-University Bochum, 32429 Minden, Germany; 4 South African College of Music, University of Cape Town, Cape Town 7700, South Africa

**Keywords:** dysphonia, laryngeal palpation, validity, reliability, voice diagnostic

## Abstract

**Background/Objectives**: Voice disorders caused by laryngeal hypertension can impact volume, quality, pitch, resonance, flexibility, and stamina. The laryngeal palpation is a tactile-perceptual assessment, which is one of a few examination methods to evaluate laryngeal hypertension. Laryngeal palpation is a manual examination of the extrinsic and paralaryngeal tissues of the larynx (e.g., lateral laryngeal mobility, thyrohyoid and cricothyroid spaces, vertical laryngeal position/mobility, and pain) through the examiner’s fingers. It can be performed during rest (static assessment) or during phonation (dynamic assessment) of the individual being evaluated. This study aimed to validate a novel laryngeal palpation tool with quantitative ordinal scores by assessing its reliability and diagnostic accuracy establishing preliminary clinical cut-off values, and examining its correlations with self-reported voice disorder symptoms. **Methods**: In a prospective, controlled validation study, 33 participants were selected to assess the validity and reliability of the novel diagnostic tool in a clinical sample and healthy controls. The clinical sample (*n* = 19) comprised individuals diagnosed with voice disorders, whereas the healthy control group (*n* = 14) included participants with no history or symptoms of voice pathology. The novel laryngeal palpation tool was employed by two independent examiners to assess both static and dynamic laryngeal function in all participants. In addition, each participant completed the following questionnaires: Voice Handicap Index (VHI-30) with the 30-item, Vocal Fatigue Index (VFI), and the Vocal Tract Discomfort Scale (VTD). **Results**: Static palpatory assessment of laryngeal tension demonstrated excellent discriminatory power between groups and tension levels (AROC = 0.979), along with high intra-rater (ICC = 0.966) and inter-rater reliability (ICC = 0.866). Significant correlations were revealed between the static palpation results and the VHI scores (r = 0.496; *p* < 0.01) and VFI (r = 0.514; *p* < 0.01). For the dynamic evaluation of the palpation tool, comparable results for the validity (AROC = 0.840) and reliability (inter-rater: ICC = 0.800, and intra-rater: ICC = 0.840) were revealed. However, no significant correlations were found between dynamic palpation and self-perceived questionnaires, although some were likely found with static palpation. The validity of the total score was found to be AROC = 0.992. **Conclusions**: The static and dynamic assessments using the novel laryngeal palpation tool demonstrated promising reliability and diagnostic accuracy, providing initial evidence to support its clinical utility. Further studies are needed to establish broader validation.

## 1. Introduction

Effective voice production relies, among others, on a complex interplay of laryngeal mechanisms including sufficient respiratory support, complete glottal closure, an intact vocal fold cover, and precise regulation of vocal fold length and tension [1]. Although further regions of the body (e.g., head position, posterior neck, jaw movement, shoulders, cervical spinous processes, spinal curve, and rib cage) may affect voice production, the larynx plays a key role of the vocal mechanism [2]. Anatomically, it comprises four key structural units: the cartilaginous framework, mucosal lining, intrinsic musculature, and extrinsic musculature. The function of the intrinsic larynx muscles are abduction, adduction, and tension of the vocal folds and these muscles are connected to the cartilages of the larynx [3]. The extrinsic laryngeal muscles are anatomically anchored, among other structures, either above or below the hyoid bone or the larynx itself, contributing to vertical laryngeal positioning and mobility. They maintain the position of the larynx in the neck by lowering or raising the larynx. Changing the position of the larynx may have consequences for the tension or angle between laryngeal cartilages and the resting length of the intrinsic muscles [2]. These muscle groups are responsible for a stable laryngeal frame to ensure the effective working of the intrinsic muscles [4]. Altered laryngeal muscular tension can therefore cause various types of dysphonia. Dysphonia is defined as an impairment in voice production—encompassing alterations in vocal quality, pitch, loudness, or phonatory effort—and/or associated reductions in voice-related quality of life [5]. These symptoms are highly prevalent, affecting approximately one-third of the population at some point during their lifespan, with presentations ranging from transient episodes to chronic conditions [5,6]. Among individuals diagnosed with a voice disorder, functional dysphonia represents one of the most frequently identified etiologies [7]. Functional voice problems are characterized by dysregulated or imbalanced laryngeal and/or paralaryngeal muscle activity causing dysphonia [8]. Functional dysphonia, also called muscle tension dysphonia [9] or malregulative dysphonia [10], can be broadly classified into primary and secondary forms: primary functional dysphonia is characterized by the absence of identifiable organic pathology, psychogenic influences, or neurological etiologies affecting voice production or laryngeal comfort, whereas secondary functional dysphonia arises in the context of coexisting mucosal disease and/or as a compensatory response to glottic insufficiency [4]. Within this framework, six distinct subtypes or severity levels have been described [11,12].

To accurately diagnose voice disorders, the use of a comprehensive test battery comprising multiple assessment modalities is recommended, structured according to the Basic Protocol of the European Laryngological Society, which can generally be categorized into five distinct domains [5,13,14]: (1) videolaryngostroboscopy, (2) perception, (3) aerodynamics, (4) acoustics, and (5) self-evaluation. Laryngeal tension is not directly mentioned in this protocol but can be assessed by instrumental methods, such as videolaryngostroboscopy (i.e., muscle tension patterns by Morrison et al. [15], and Morrison and Rammage [11]), surface electromyography (sEMG), or laryngeal electromyography (LEMG). Palpation is among the most widely utilized methods for evaluating laryngeal muscle tension owing to its simplicity, time- and cost-efficiency, and the absence of associated adverse effects [16,17]. It belongs to the tactile perception category assessment and provides information about the laryngeal function and the degree of muscular tension using the hands to assess the patient at rest without phonation (static assessment) and during phonation, such as on the sustained vowel /a/ at comfortable pitch and loudness (dynamic assessment) [17]. Examiners palpate with thumb and forefinger (or middle finger) to evaluate, for example, lateral laryngeal mobility, the thyrohyoid and cricothyroid spaces (including narrowing/elasticity), vertical laryngeal position/mobility, and pain on prompt. This is a method to document visible or palpatory laryngeal muscle tension with a focus on the extrinsic laryngeal muscles, without the use of invasive instruments such as endoscopic guidance. The examiner should possess a range of palpatory skills to evaluate joint mobility, the quality of perijoint soft tissues, and the function of muscles acting across the joint [18]. In this context, Lieberman described four key palpatory criteria (tissue texture, asymmetry, restriction of motion, and tenderness (TART)), which form the basis of the Osteopathic Manipulative Treatment TART protocol and are consistent with the training of the two speech-language pathologists (SLTs) who performed the present assessments. These criteria are reflected in the structured schema of our palpation tool (see Supplementary Table S1). Importantly, when assessing the thyrohyoid and cricothyroid spaces, not only muscular but also elastic and perijoint soft tissues must be considered, as they contribute substantially to laryngeal mobility and tension regulation.

Palpation tools published between 1980 and 2013 exhibited certain weaknesses in various aspects of reliability and validity [16]. For example, inter-rater and intra-rater reliability results in palpation instruments were almost never analyzed, the number of examiners and number of participants in studies was usually low, and no investigations of concurrent validity regarding LEMG or the low number of concurrent validity studies between palpation instruments and X-ray imaging variants or sEMG were considered [16]. The examiner’s skills, as well as the clarity and precision of the descriptions or assessment scores for the palpated areas (e.g., quantitative vs. qualitative results), may influence the outcomes of palpatory assessments. During palpatory assessments of the paralaryngeal muscles, the thyrohyoid space, lateral resistance, dynamic laryngeal elevation or depression, and the suprahyoid muscles are regarded as the most reliable and valid areas [16,17]. Based on this knowledge Barsties and Thede [17] developed a new and systematic laryngeal palpation tool. The principal modifications compared to existing palpation tools included the implementation of a quantitative rating scale with clearly defined and easily distinguishable evaluation criteria for specific palpation regions, the separate consideration of static and dynamic assessment components, and the incorporation of examiner-friendly palpatory parameters—such as thyrohyoid space, lateral resistance, and dynamic laryngeal mobility—based on the framework proposed by Khoddami et al. [16].

The primary aim of this study was to evaluate the validity and reliability of a novel laryngeal palpation tool designed to assess both static and dynamic palpatory parameters in individuals with and without voice disorders. Additionally, the study sought to establish preliminary cut-off values to facilitate the integration of this tool into clinical practice. A secondary objective was to examine the correlations between palpatory findings and self-reported vocal health and voice disorder symptoms, as measured by three standardized questionnaires.

## 2. Materials and Methods

### 2.1. Study Design

A prospective, controlled diagnostic validation study was performed to assess the psychometric properties (validity and reliability) of the newly developed diagnostic tool. The study included a clinical group of patients with a confirmed diagnosis and a demographically matched healthy control group. Over a three-month period at various locations at the SRH University and University of Münster, the study aimed to evaluate the effectiveness of the new laryngeal palpation tool in voice diagnostics. The study was approved by the Ethics Committee of the German Federal Association for Academic Speech Therapy and Logopedics (Deutscher Bundesverband für akademische Sprachtherapie und Logopädie, dbs; reference number 23-TEMP922599-KA-ESpK) and registered at the German Clinical Trials Register (ID: DRKS00032866).

### 2.2. Participants

Participants were categorized based on medical vocal health status into a vocally healthy group (*n* = 14) and two dysphonia groups (functional voice disorder: *n* = 12 and organic voice disorder: *n* = 7). Thus, a total of 33 adults were included. Inclusion criteria for vocally healthy participants comprised the absence of laryngitis within the preceding three months and scores indicative of normal vocal function on the German versions of three standardized self-assessment instruments: the 30-item Voice Handicap Index (VHI), the Vocal Fatigue Index (VFI), and the Vocal Tract Discomfort (VTD) scale. A healthy voice was defined as scores falling below established thresholds on standardized instruments: Voice Handicap Index (VHI) score < 15 [19], Vocal Fatigue Index (VFI) cluster 1 score < 17.5 [20], and Vocal Tract Discomfort (VTD) score < 14 [21,22]. Additional exclusion criteria for this group included any history of voice therapy for a diagnosed voice disorder and it was ensured that none of the participants had a history of thyroid or other neck surgery. In addition to fulfilling the questionnaire-based cut-off criteria, the absence of voice disorders was confirmed by a structured anamnesis. No additional instrumental examinations (e.g., videostroboscopy or acoustic analysis) were performed, as the study design aimed to validate the palpation tool within a pragmatic clinical framework.

Participants in the dysphonia groups were allocated following a comprehensive clinical phoniatric evaluation confirming the presence of a voice disorder. At the time of assessment, participants were either not receiving voice therapy or were in the initial stages of intervention. Exclusion criteria included a history of radiochemotherapy involving the cervical region, a diagnosis of osteoporosis or fibromyalgia, or age under 18 years. Further exclusion criteria were a history of laryngeal trauma or psychological trauma related to voice use, and persons who were or were considered legally incapacitated. Although routine phoniatric examination including videostroboscopy was performed in the dysphonia group, these findings were not systematically recorded or analyzed, since the present study focused on extrinsic and paralaryngeal muscle tension assessed through palpation.

All participants completed the VHI, VFI, and VTD questionnaires to provide further information about their self-perceived vocal health status and symptoms of voice disorder (see Table 1).

### 2.3. Palpation Tool

The palpation tool established by Barsties and Thede [17] was used, which is a standardized external manual palpation protocol for the tactile perceptual evaluation of the larynx. The protocol of this tool is provided in the Supplementary Table S1. The assessment procedure comprised both static and dynamic evaluations of laryngeal mobility, the thyrohyoid space, and the cricothyroid space, with specific attention to movement restrictions, structural narrowing, and participant-reported pain. The tool aims to quantify changes in muscular tension and to provide a comprehensive understanding of laryngeal function in relation to muscle tension state. For clinical utility, it is important to consider both static and dynamic evaluations of laryngeal mobility, as there may be inherent variability in phonation across repetitions, differences in muscle recruitment patterns during voicing, and the potential masking of underlying tension patterns by compensatory movements during speech production.

During palpation, the participants sat upright with a neutral head position (i.e., no raising, depression, retraction, or protrusion of the chin). The examiner used the thumb and forefinger or middle finger for all palpated areas, starting with the static (without phonation) assessment. Palpation focused on the lateral laryngeal mobility, thyrohyoid space, and cricothyroid space. The participants were asked during each cluster of palpation whether they experienced pain (excluding discomfort, unfamiliar sensations, or strangeness). Pain during palpation was evaluated immediately after each examined region using a verbal yes/no response. The dynamic assessment followed, during which the participant sustained the vowel [a:] at a comfortable pitch and loudness. This assessment included the larynx/hyoid position along with the thyrohyoid and cricothyroid spaces. The participants were again asked during each cluster about the presence of pain, considering the same exclusion criteria as in the static assessment.

Cluster ratings were calculated based on findings within each area. Each cluster has subscores linked to specific parameters, except for the pain cluster, where all pain findings contribute to a single subscore. Subscores were summed to determine the total static assessment score (SA-T) and total dynamic assessment score (DA-T). Additionally, SA-T and DA-T were combined to form an overall total score (TS), to provide a comprehensive assessment.

The standardized palpation was performed by two speech-language pathologists. Examiner 1 performed the first assessment using the palpation tool. Subsequently, examiner 2 conducted a full examination. In the third step, examiner 1 repeated the static assessment, while examiner 2 repeated the dynamic assessment. To minimize bias, the examiners were blinded to each other’s findings throughout the process.

### 2.4. Statistics

Statistical analyses were completed using SPSS for Windows version 29.0.1.0 (IBM Corp., Armonk, NY, USA). The analysis of the study focused on inter-rater and intra-rater reliability, validity of the palpation results, and correlations between the palpation results and self-evaluation using questionnaires. Dynamic and static palpation assessments were considered separately.

First, the rater reliability was calculated using Intraclass Correlation Coefficient (ICC) for single measures. This ICC is a standard statistical method used to indicate the strength of reliability among quantitative assessments from individual examiners, accounting for both correlation and differences. The ICC ranges from 0.00 (indicating no reliability) to 1.00 (indicating perfect reliability). For interpreting ICC values, a common guideline suggests that an ICC above 0.75 indicates good reliability [23]. For reliability to be considered strong, the ICC value should ideally exceed 0.90 [23].

Second, to determine the validity of the new laryngeal palpation tool, the receiver operating characteristic (ROC) curve analysis was used with the aim of assessing the diagnostic predictive accuracy of the palpation tool for discriminating between the vocally healthy group and the dysphonia group. An area under the ROC curve (AROC) of 1.0 indicates perfect classification between normal tension and hypertension, while an AROC of 0.5 reflects chance-level diagnostic accuracy [23]. Sensitivity, specificity, and likelihood ratios (positive and negative results; LR+/LR−) for the palpation outcomes were calculated using ROC curve analysis [24]. The Youden index was applied to identify preceding optimal threshold levels for SA-T and DA-T palpation outcomes. The coordinates of the ROC curve were used to calculate the Youden index to establish the cut-off values.

Third, to evaluate the association between the palpation outcomes and self-reported questionnaire scores, Pearson correlation coefficients were calculated and interpretation guidelines were provided by Frey et al. [25]; *p*-values less than 0.05 were indicative of statistical significance.

## 3. Results

The intra-rater reliability analysis yielded an intraclass correlation coefficient (ICC) of 0.97 (95% CI: 0.933–0.983) for the static assessment and 0.84 (95% CI: 0.701–0.918) for the dynamic assessment, indicating high levels of agreement and acceptable intra-rater reliability. Similarly, inter-rater reliability demonstrated comparable results, with an ICC = 0.87 for static assessment (SA-T) and 0.80 for the dynamic assessment (DA-T).

**Figure 1:**
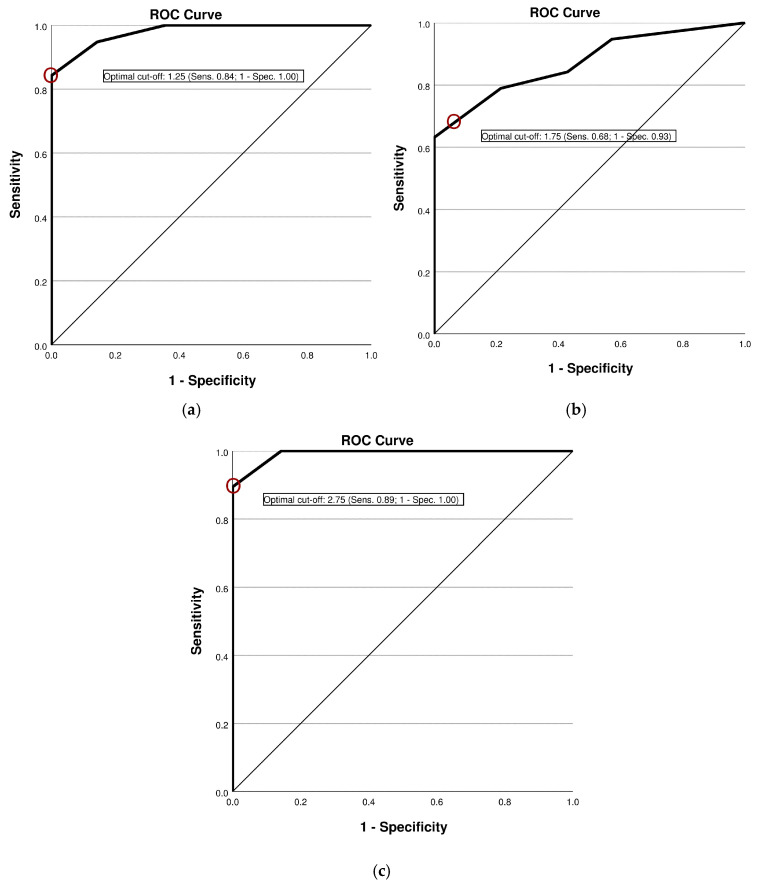
Receiver operating characteristic curve illustrating diagnostic accuracy of the palpation tool: (**a**) Results from the SA-T; (**b**) results from the DA-T; (**c**) results from the TS.

The diagnostic accuracy of the new laryngeal palpation tool in differentiating between vocally healthy volunteers and individuals with (para-)laryngeal hypertension is summarized in Table 2. For both components of the palpation assessment (SA-T and DA-T), the AROC statistics indicated acceptable discriminatory power in distinguishing muscular tension differences between the two groups (see Figure 1 and Table 2). For both palpation assessment parts, a preceding clinical cut-off score <2 was found to present the best balance between sensitivity and specificity according to the Youden index. For assessments incorporating the total score (TS), a cut-off value of 2.75 is recommended as it provides an evidence-based threshold based on the Youden index. Sample scores from the palpation sheets of a typical healthy patient and a dysphonic participant are presented in Supplementary Table S2.

The relationships between SA-T and DA-T and the VHI, VFI-cluster 1, and VTD were analyzed using Pearson’s correlation. Significant correlations were found between the static palpation assessment and the total score for both palpation assessments with the VHI (*r* = 0.496, *p* < 0.01 and *r* = 0.416, *p* < 0.05, respectively) and VFI-cluster 1 (*r* = 0.514, *p* < 0.01 and *r* = 0.451, *p* < 0.01, respectively). No significant correlations were identified between the dynamic palpation assessment and the three questionnaires. Pain was qualitatively reported by some participants during palpation, but no standardized pain scale was applied. Therefore, no conclusions can be drawn about the prevalence of pain in organic dysphonia based on our data.

## 4. Discussion

This study investigated the reliability and validity of a novel laryngeal palpation tool in a cohort of 33 participants, including individuals with and without clinically diagnosed voice disorders. The tool demonstrated high reliability, as reflected by an intraclass correlation coefficient (ICC) of 0.97 for intra-rater and 0.87 for inter-rater reliability, indicating both a consistent application within examiners and strong agreement between examiners. To assess diagnostic validity, a receiver operating characteristic (ROC) curve analysis was carried out, yielding an AROC of 0.979, indicative for excellent discriminatory power. A cut-off score of 1.25 allowed for the reliable differentiation of healthy voices from voice disorders, with a sensitivity of 84.2% and a specificity of 100%. These findings suggest that static palpation reliably identifies pathological voice conditions while minimizing false positives. In this sample, participants with organic dysphonia tended to show higher palpation scores than those with functional dysphonia. However, this exploratory observation must be interpreted with caution, as the sample size was limited, group distributions were unbalanced with respect to age and gender, and no repeated measurements over time were available. Future studies with larger and stratified cohorts are required to investigate potential differences between dysphonia subtypes.

At the level of subjective assessments, a significant correlation was found between the palpation results and the values of the Voice Handicap Index (VHI) and the Voice Fatigue Index (VFI, part 1). Laryngeal palpation thus evidently captures aspects of vocal function that are also subjectively perceived by the patients themselves. However, the lack of correlation with the information in the Vocal Tract Discomfort Scale (VTD) indicates that sensory discomfort in the throat area does not necessarily correspond to the palpable muscle tension.

In addition to static assessment, dynamic laryngeal palpation was conducted to evaluate muscular function during phonation. Initial analysis using the Youden index identified an optimal cut-off value of 2.25, yielding a specificity of 100% and a sensitivity of 63.2%. However, given the use of whole-point scoring in the palpation tool and the observed mean score in the dysphonia group (M = 2.55; SD = 1.4), a revised cut-off value of 1.75 was deemed more clinically appropriate. This adjusted threshold improved sensitivity to 68.4% while maintaining a high specificity of 92.9%, thereby enhancing the tool’s ability to detect voice disorders. In addition, the positive likelihood ratio (LR+) of 9.63 at this cut-off reflects a near-excellent level of diagnostic accuracy. It should be emphasized that static and dynamic palpation assessments are complementary rather than interchangeable; a normal result in one does not preclude abnormalities in the other. This underscores the value of incorporating both assessments into a comprehensive clinical evaluation.

Preliminary analyses indicate that the TS enhances the overall discriminatory power of the assessment, capturing both static and dynamic manifestations of muscle dysfunction. This composite measure supports a more nuanced clinical decision-making process, as it demonstrates the highest ratio of sensitivity (89.5%) to specificity (100%).

Beyond the primary diagnostic performance metrics, further analyses support the clinical relevance of the palpation tool. Significant correlations between static palpation scores and the Voice Handicap Index (VHI), as well as the Vocal Fatigue Index (VFI), suggest that the palpatory method captures perceptible aspects of vocal dysfunction as reported by patients. This alignment between objective and subjective measures reinforces the potential utility of static palpation not only in initial diagnosis but also in monitoring patient-reported outcomes over time and tracking therapeutic progress. Conversely, the lack of correlation between palpation results and the Vocal Tract Discomfort Scale (VTD) scores indicates that perceived laryngeal discomfort may not directly reflect measurable muscular tension, highlighting the importance of including both physical examination and self-report measures in clinical assessments. Differences in palpation scores between functional and organic dysphonia could reflect underlying physiological factors. In organic dysphonia, structural changes in the vocal folds or surrounding muscles might lead to increased or asymmetric tension detectable by palpation, whereas functional dysphonia may lack structural pathology, resulting in more variable tension patterns. Furthermore, the higher palpation scores observed in participants with organic dysphonia relative to those with functional dysphonia could reflect compensatory muscular over-activation or rigidity associated with structural pathology. This suggests that static palpation may be particularly sensitive to secondary tension patterns in organic voice disorders and could aid in differential diagnostic considerations. Additionally, the divergence between static and dynamic palpation outcomes underscores the complementary nature of both assessments: while static palpation likely reflects baseline muscular tension, dynamic palpation may capture task-specific dysfunctions arising during phonation. The absence of strong correlations between dynamic palpation and self-reported measures could indicate that dynamic muscular tension is not always consciously perceived by patients, further supporting the integration of both static and dynamic assessments in a comprehensive diagnostic protocol. Finally, although the selected cut-off values demonstrated sufficient sensitivity and specificity, their clinical applicability should be validated in larger, more demographically diverse samples to confirm their generalizability across different age groups and dysphonia subtypes.

Although the sample size was small (*n* = 33), the defined eligibility criteria support internal validity. However, age differences among groups may have influenced the results, and future studies should ensure closer age-matching. Of the study group, 12 participants belonged to the vocally healthy cohort (M = 37.07 years, SD = 16.55), whereas the dysphonia group was older on average (M = 57.0 years, SD = 19.3). This disparity likely reflects the recruitment of vocally healthy individuals from a speech therapy training program with predominantly younger trainees. Nevertheless, the comparable standard deviations suggest a relatively homogeneous age distribution across groups.

Despite the promising findings regarding the diagnostic reliability and validity of the laryngeal palpation tool, several limitations must be acknowledged. First, the overall sample size was modest, and the demographic composition was imbalanced, with the vocally healthy group primarily consisting of younger speech therapy trainees and the dysphonia group exhibiting a higher mean age. This discrepancy may limit the generalizability of the results across broader populations. Second, the dysphonia group included both organic and functional voice disorders without further stratification by specific etiologies, despite the likelihood that muscular tension patterns vary across subtypes.

Third, the study employed only two trained speech-language pathologists as examiners, which, although yielding high reliability, may not reflect the inter-examiner variability encountered in typical clinical settings. Fourth, the study followed a single-session design, precluding an assessment of test–retest reliability over time and limiting conclusions about the temporal stability of the tool. Additionally, examiner blinding was not implemented, introducing potential bias, and the repeated dynamic palpation was not accompanied by the re-administration of static palpation, which restricts the ability to directly compare repeated measures.

A procedural inconsistency arose in the second session, where one examiner conducted only static palpation and the other only dynamic palpation. This division, driven by time constraints and thesis requirements—with each examiner focusing on a different palpation component—allowed for methodological depth but may have introduced systematic bias, limiting intra-examiner reliability and warranting clarification in the methodology or discussion. Furthermore, although participants were asked to report pain during palpation, no standardized pain scale (e.g., visual analog scale or Likert scale) was used, which may have introduced subjective variability and limited reproducibility. Given that discomfort was explicitly excluded during the palpation procedure, the lack of correlation with the VTD scale is not surprising and should be interpreted with caution. Future studies should therefore incorporate validated pain measures to enhance comparability and objectivity.

Another important limitation is the absence of comparison with an established instrumental gold standard, such as surface electromyography (sEMG), laryngeal EMG, or imaging-based techniques, which could have provided an objective benchmark for validating palpation findings. Lastly, as the development and validation of the palpation tool were conducted by the same research group, the possibility of confirmation bias cannot be entirely excluded, despite adherence to standardized procedures. These limitations highlight the need for future studies with larger, more heterogeneous samples, examiner blinding, independent validation, and the integration of objective reference measures to further establish the clinical robustness of the tool.

Despite these limitations, the findings offer valuable insights into the clinical use of structured laryngeal palpation. Using standardized protocols, the tool showed strong reliability and meaningful cut-off values, supporting its role in distinguishing healthy from dysphonic individuals. Its ability to detect muscle tension patterns aligned with patient-reported symptoms highlights its diagnostic and monitoring potential. Future research should include larger, more diverse cohorts and differentiate dysphonia subtypes to refine diagnostic accuracy. Incorporating longer assessment intervals and engaging independent examiners could further mitigate potential bias and enhance generalizability. Moreover, integrating objective reference standards such as electromyography or imaging-based techniques would provide additional construct validity and contribute to a more comprehensive understanding of laryngeal muscle tension in both functional and organic disorders.

The present study should be regarded as a first step toward a broader validation of the palpation tool. While the results support its potential utility, the limitations in sample size, group heterogeneity, and lack of objective reference measures preclude definitive validation. Future research must therefore confirm these findings in larger and more stratified cohorts. Given the lack of validated palpation methods, these findings are especially relevant. With high reliability and empirically derived cut-off values, the study provides a basis for clinical integration. The observed correlation between palpation scores and self-reported symptoms indicates that the method reflects both objective dysfunction and patient experience, supporting its use alongside existing diagnostic tools in a more integrated approach.

## 5. Conclusions

This study provides the first evidence that the newly developed laryngeal palpation tool is a reliable and diagnostically accurate method for assessing muscular tension in individuals with and without voice disorders. The present findings support its potential integration into clinical voice diagnostics, but further investigation is necessary. Nevertheless, the tool enables effective differentiation between healthy and dysphonic voices, with static palpation scores significantly correlating with self-reported measures of vocal handicap and fatigue. These findings support the integration of manual laryngeal palpation into clinical voice diagnostics as a complementary measure, alongside existing perceptual, acoustic, and instrumental methods. Based on the present findings, this study advocates for the use of the total score (TS) as an integrative metric in clinical assessment with a cut-off value of 2.75. Notably, this study provides a strong foundation for considering structured laryngeal tension assessment as a potential addition to the perception pillar in a future revision of, for example, the Basic Protocol of the European Laryngological Society of voice assessment. By contributing both empirical data and a standardized implementation framework, the present work lays important groundwork for advancing the multidimensional evaluation of voice disorders within interdisciplinary clinical settings.

## Figures and Tables

**Table 1:** Descriptive statistics of all participants regarding gender, age and questionnaire results.

Group (Number of Participants)	Gender	Age Mean (SD)	VHI Mean and (SD)	VFI-Cluster 1 Mean (SD)	VTD Mean (SD)
Vocally healthy (14)	M: 6 F: 8	37.07 years (±16.55 years)	3.71 (±3.20)	3.21 (±2.83)	4.29 (±3.89)
Dysphonia (19) with functional- (12) and organic (7) voice disorders	M: 4 F: 15	57.00 years (±19.30 years)	47.16 (±32.68)	27.58 (±16.81)	23.89 (±20.75)

**Table 2:** Diagnostic accuracy of the new laryngeal palpation tool.

Part of the Palpation Assessment	Vocally Healthy Group Mean (SD)	Dysphonia Group Mean (SD)	AROC	Cut-Off	Sensitivity	Specificity	LR+	LR−
Static assessment (SA-T)	0.25 (± 0.38)	2.26 (± 1.03)	0.979	1.25	84.2%	100%	∞	0.158
Dynamic assessment (DA-T)	0.64 (± 0.69)	2.55 (± 1.54)	0.872	1.75	68.4%	92.9%	9.63	0.340
Total score (TS)	0.89 (± 0.79)	4.82 (± 1.71)	0.992	2.75	89.5%	100%	∞	0.105

## Data Availability

The original contributions presented in the study are included in the article; further inquiries can be directed to the corresponding author.

## Supplementary Materials

The following supporting information can be downloaded at: https://www.mdpi.com/article/10.3390/jcm14176309/s1, Table S1: Protocol of the new laryngeal palpation tool; Table S2: Palpation sheets of sample scores of a typical healthy and a dysphonic participant.
