# Supplementary material for: The Reliability and Validity of a New Laryngeal Palpation Tool for Static and Dynamic Examination
Source: J Clin Med. 2025 Sep 6;14(17):6309. doi: 10.3390/jcm14176309 (PMC12428919; doi:10.3390/jcm14176309)
Supplement: Supplementary file 1 [file jcm-14-06309-s001.zip › jcm-3765584-supplementary.pdf]

TABLE S1 Palpation tool

Part 1. Laryngeal palpation instrument: (1) Investigation at rest (static assessment)

Cluster	Parameter	Score	Subtotal score	Total score of SA
Laryngeal lateral mobility	No resistance	0	0-2	0-8
	Minimal resistance towards the end of the movement	1		
	Highly lateral resistance	2		
Thyrohyoid space	Space bilateral equal	0	0-2	
	Space bilateral unequal	1		
	Space not perceptible (closed space)	2		
Cricothyroid space	Space perceptible	0	0-1	
	Space not perceptible (closed space)	1		
Pain (asked to the patient during palpation)	No pain	0	0-3	
	Pain at laryngeal lateral mobility	1		
	Pain at thyrohyoid space	1		
	Pain at cricothyroid space	1		

Part 2. Laryngeal palpation instrument: (2) Investigation during phonation (dynamic assessment)

Cluster	Parameter	Score	Subtotal score	Total score of DA
Position of the larynx/hyoid	Stays at equal position	0	0-2	0-9
	Laryngeal elevation	1		
	Laryngeal depression	1		
Thyrohyoid space	Space stays comparable as in rest	0	0-2	
	Space smaller than at rest (unilateral)	1		
	Space smaller than at rest (bilateral)	2		
Cricothyroid space	Space stays perceptible	0	0-2	
	Space not perceptible (closed space)	1		
	Space smaller than at rest	2		
Pain (asked to the patient during palpation)	No pain	0	0-3	
	Pain at laryngeal mobility	1		
	Pain at thyrohyoid space	1		
	Pain at cricothyroid space	1		

Table S2 An example of sample scores from the palpation sheet of a typical healthy and a voice-disordered participant

Palpation sheet of a typical healthy participant
Examiner 1 in blue
Examiner 2 in red

First and second Round:

Part 1. Laryngeal palpation instrument: (1) Investigation at rest (static assessment)

Cluster	Parameter	Score	Subtotal score	Total score of SA
Laryngeal lateral mobility	No resistance	0	0-2<br>0<br>0	0-8<br>1<br>1
	Minimal resistance towards the end of the movement	1		
	Highly lateral resistance	2		
Thyrohyoid space	Space bilateral equal	0	0-2<br>1<br>1	
	Space bilateral unequal	1		
	Space not perceptible (closed space)	2		
Cricothyroid space	Space perceptible	0	0-1<br>0<br>0	
	Space not perceptible (closed space)	1		
Pain (asked to the patient during palpation)	No pain	0	0-3<br>0<br>0	
	Pain at laryngeal lateral mobility	1		
	Pain at thyrohyoid space	1		
	Pain at cricothyroid space	1		

Part 2. Laryngeal palpation instrument: (2) Investigation during phonation (dynamic assessment)

Cluster	Parameter	Score	Subtotal score	Total score of DA
Position of the larynx/hyoid	Stays at equal position	0	0-2<br>0<br>0	0-9<br>0<br>0
	Laryngeal elevation	1		
	Laryngeal depression	1		
Thyrohyoid space	Space stays comparable as in rest	0	0-2<br>0<br>0	
	Space smaller than at rest (unilateral)	1		
	Space smaller than at rest (bilateral)	2		
Cricothyroid space	Space stays perceptible	0	0-2<br>0<br>0	
	Space not perceptible (closed space)	1		
	Space smaller than at rest	2		
	No pain	0	0-3	

Pain (asked to the patient during palpation)	Pain at laryngeal mobility	1	0<br>0	
	Pain at thyrohyoid space	1		
	Pain at cricothyroid space	1		

Third and fourth Round:

Part 1. Laryngeal palpation instrument: (1) Investigation at rest (static assessment)

Cluster	Parameter	Score	Subtotal score	Total score of SA
Laryngeal lateral mobility	No resistance	0	0-2<br>0	0-8<br>1
	Minimal resistance towards the end of the movement	1		
	Highly lateral resistance	2		
Thyrohyoid space	Space bilateral equal	0	0-2<br>1	
	Space bilateral unequal	1		
	Space not perceptible (closed space)	2		
Cricothyroid space	Space perceptible	0	0-1<br>0	
	Space not perceptible (closed space)	1		
Pain (asked to the patient during palpation)	No pain	0	0-3<br>0	
	Pain at laryngeal lateral mobility	1		
	Pain at thyrohyoid space	1		
	Pain at cricothyroid space	1		

Part 2. Laryngeal palpation instrument: (2) Investigation during phonation (dynamic assessment)

Cluster	Parameter	Score	Subtotal score	Total score of DA
Position of the larynx/hyoid	Stays at equal position	0	0-2<br>0	0-9<br>1
	Laryngeal elevation	1		
	Laryngeal depression	1		
Thyrohyoid space	Space stays comparable as in rest	0	0-2<br>1	
	Space smaller than at rest (unilateral)	1		
	Space smaller than at rest (bilateral)	2		
Cricothyroid space	Space stays perceptible	0	0-2<br>0	
	Space not perceptible (closed space)	1		
	Space smaller than at rest	2		
	No pain	0	0-3	

Pain (asked to the patient during palpation)	Pain at laryngeal mobility	1	0	
	Pain at thyrohyoid space	1		
	Pain at cricothyroid space	1		

Palpation sheet of a voice-disordered participant
Examiner 1 in blue
Examiner 2 in red

First and second Round:

Part 1. Laryngeal palpation instrument: (1) Investigation at rest (static assessment)

Cluster	Parameter	Score	Subtotal score	Total score of SA
Laryngeal lateral mobility	No resistance	0	0-2<br>2<br>2	0-8<br>2<br>3
	Minimal resistance towards the end of the movement	1		
	Highly lateral resistance	2		
Thyrohyoid space	Space bilateral equal	0	0-2<br>0<br>1	
	Space bilateral unequal	1		
	Space not perceptible (closed space)	2		
Cricothyroid space	Space perceptible	0	0-1<br>0<br>0	
	Space not perceptible (closed space)	1		
Pain (asked to the patient during palpation)	No pain	0	0-3<br>0<br>0	
	Pain at laryngeal lateral mobility	1		
	Pain at thyroyoid space	1		
	Pain at cricothyroid space	1		

Part 2. Laryngeal palpation instrument: (2) Investigation during phonation (dynamic assessment)

Cluster	Parameter	Score	Subtotal score	Total score of DA
Position of the larynx/hyoid	Stays at equal position	0	0-2<br>0<br>0	0-9<br>4<br>3
	Laryngeal elevation	1		
	Laryngeal depression	1		
Thyrohyoid space	Space stays comparable as in rest	0	0-2<br>2<br>2	
	Space smaller than at rest (unilateral)	1		
	Space smaller than at rest (bilateral)	2		

Cricothyroid space	Space stays perceptible	0	0-2<br>2<br>1	
	Space not perceptible (closed space)	1		
	Space smaller than at rest	2		
Pain (asked to the patient during palpation)	No pain	0	0-3<br>0<br>0	
	Pain at laryngeal mobility	1		
	Pain at thyrohyoid space	1		
	Pain at cricothyroid space	1		

Third and fourth Round:

Part 1. Laryngeal palpation instrument: (1) Investigation at rest (static assessment)

Cluster	Parameter	Score	Subtotal score	Total score of SA
Laryngeal lateral mobility	No resistance	0	0-2<br>2	0-8<br>2
	Minimal resistance towards the end of the movement	1		
	Highly lateral resistance	2		
Thyrohyoid space	Space bilateral equal	0	0-2<br>0	
	Space bilateral unequal	1		
	Space not perceptible (closed space)	2		
Cricothyroid space	Space perceptible	0	0-1<br>0	
	Space not perceptible (closed space)	1		
Pain (asked to the patient during palpation)	No pain	0	0-3<br>0	
	Pain at laryngeal lateral mobility	1		
	Pain at thyrohyoid space	1		
	Pain at cricothyroid space	1		

Part 2. Laryngeal palpation instrument: (2) Investigation during phonation (dynamic assessment)

Cluster	Parameter	Score	Subtotal score	Total score of DA
Position of the larynx/hyoid	Stays at equal position	0	0-2<br>0	0-9<br>4
	Laryngeal elevation	1		
	Laryngeal depression	1		
Thyrohyoid space	Space stays comparable as in rest	0	0-2<br>2	
	Space smaller than at rest (unilateral)	1		
	Space smaller than at rest (bilateral)	2		

Cricothyroid space	Space stays perceptible	0	0-2	
	Space not perceptible (closed space)	1	2	
	Space smaller than at rest	2		
Pain (asked to the patient during palpation)	No pain	0	0-3	
	Pain at laryngeal mobility	1	0	
	Pain at thyrohyoid space	1		
	Pain at cricothyroid space	1		
